# Validation of a patient-based strategy for ambulatory monitoring of oncological pain by a mobile application

**DOI:** 10.1038/s41598-025-34057-5

**Published:** 2026-02-02

**Authors:** Marina Castillo, Julia Casado-Gómez-Pallete, Jorge Lázaro-Bailón, José M. Iniesta-Chamorro, M. Laura García-Bermejo, Sonsoles Sancho, M. Elena Hernando, Carolina de la Pinta

**Affiliations:** 1 Biomedical Engineering and Telemedicine Centre, ETSI Telecomunicación, Center for Biomedical Technology, Universidad Politécnica de Madrid, Madrid, Spain; 2 Hospital Universitario Ramón y Cajal, IRYCIS, Madrid, Spain; 3 Biomarkers and Therapeutic Targets Group and Core Facility, Ramón y Cajal Health Research Institute (IRYCIS), Madrid, 28034 Spain; 4 CIBER de Bioingeniería, Biomateriales y Nanomedicina, Instituto de Salud Carlos III, Madrid, Spain

**Keywords:** Telemedicine, Pain monitoring, Radiotherapy, Cancer, Quality of life, Symptoms, Cancer, Diseases, Health care, Medical research, Oncology

## Abstract

The purpose of this study is to assess clinical feasibility and patient adherence to recording pain, symptoms, and medication through the mobile application Accompain. Secondary outcomes included pain evolution, quality of life, pain interference, symptom progression, medication changes, and pain alerts. In this prospective clinical validation study, adult patients with cancer-related pain used a mobile device to log pain intensity, symptoms, and daily rescue medication. The system alerted clinicians to patients with elevated pain or a high number of rescue medications. Eighty-seven patients with different tumors were recruited (60.92% women; median age 63 years; range 27–94). Data recording adherence was 73.56%. Compared to baseline (5.49 ± 1.86), patients reported decreased pain 30 days (3.79 ± 2.48, *p* < .0001), 45 days (4.18 ± 2.42, *p* < .0001), and 60 days (3.82 ± 2.37, *p* < .0001) after starting treatment. Patients with localized tumors reported poorer health and quality of life than those with metastasis (*p* < .0001). Asthenia was the most frequent symptom. Daily rescue medication reporting increased at 46–60 days after inclusion (*p* < .01). Physicians received a mean of 2.53 ± 3.89 alerts per week during the study. Ambulatory pain monitoring is feasible and allows remote assessment of patients’ clinical status, which could improve pain management and overall care quality. In our study, gender and metastatic stage are the factors that most influence treatment adherence.

## Introduction

30% of oncology patients experience pain at the time of diagnosis, and this prevalence increases to 80% in the final stages of the disease^1^. Oncologic pain monitoring primarily occurs during medical visits, where the patient’s condition is evaluated, and adjustments to analgesic therapy are made accordingly. The intensity of pain varies among patients and is measured using patient-reported scales such as the Visual Analog Scale (VAS) or the Brief Pain Inventory (BPI). These scales assess the degree of pain at specific moments and evaluate the effectiveness of analgesic treatment^2^. Furthermore, the severity of pain is assessed based on its impact on daily life, and quality-of-life questionnaires are valuable tools for this purpose^3^.

One of the manifestations of pain in oncology is breakthrough pain, characterized by incident onset (within 1 to 5 min), high intensity, and occurring with a frequency of 1 to 4 episodes per day with an average duration of 45 minutes^4^. Breakthrough pain includes end-of-dose pain and incident (rapid onset) pain due to a controllable (e.g. movement) or uncontrollable (e.g. cough or hiccups) trigger. Breakthrough pain requires the administration of rescue analgesic medications at the patient’s request. Healthcare professionals must adjust analgesic therapy based on the intensity of the patient’s pain and the need for rescue analgesics to control breakthrough pain events^5^. However, response time is constrained by the frequency of face-to-face physician visits. Increasing frequency of visits would anticipate the treatment changes, but this is not always feasible in resource-constrained scenarios. Information and communication technologies can serve as an alternative for optimizing healthcare resources, if they provide the necessary information for appropriate decision-making.

Numerous studies on pain monitoring in oncology patients are available in both the adult^6–16^ and pediatric^17,18^ literature. These studies assess the increase of pain data recording^7,14,17^ and drug administration^7,14,17^, the efficacy of pain control^6,14,17,19,20^, and the adherence to pain recording protocols^6,7,13–15,20,21^ or the use of telemedicine tools^6,13–15,20,21^. The most common pain assessment scales are Brief Pain Inventory (BPI)^6,13–15,18,19^, Memorial Symptom Assessment Scale (MSAS)^17,19^, and Numeric Rating Scale (NRS)^7,8,10,11,22^. From the perspective of healthcare professionals, communication with patients to gather information may not exist^9,12,16,17^, or it may be via phone call^10,11,13,18,20,21^, videoconference^6,13,15^, or text message^7,8,13,21,22^.

In certain instances, monitoring systems exhibit proactive behavior, assisting professionals by promptly generating alerts in cases where: MSAS 7–12 overall pain rating ≥ 7.5, or MSAS 8–18 overall pain rating ≥ 7.5^17^; 3 consecutive pain reports of ≥ 3/10^18^; baseline pain > 5 or vomiting/nausea for two consecutive days, more than four episodes of breakthrough pain in 1 day, or breakthrough pain that lasts for over 90 min^10^. ; NRS pain score exceeding 5^11^; inadequate symptom improvement, nonadherence to medication, side effects, or suicidal ideation^14^, and poorly controlled symptoms meeting preset thresholds (≥ 4/10)^20^.

After analyzing the state-of-the-art literature, it is observed that the available studies present a diverse range of sample sizes (from 11^7^ to 405^14^ patients), and the recruited populations are heterogeneous, including a wide spectrum of pathological conditions^6–11,14–18,20^. In general, the recording and reporting of rescue analgesia are limited due to the insufficient number of cases included in the studies or the short follow-up periods.

This paper describes the validation of a home monitoring strategy supported by a mobile application that assists patients in recording pain by facilitating patient-physician communication through validated questionnaires. The study assesses the evolution of pain, the adherence to the application’s usage and the evolution of quality of life.

## Methods

### Study design

A prospective, single-arm clinical study was conducted. The objective was to evaluate the effectiveness of a tool (mobile application) designed for pain monitoring. The study was approved by the Investigation Ethics Committee (code 338/21) of the Hospital Universitario Ramón y Cajal de Madrid (HURyC). The study was conducted in accordance with the guidelines for research involving human subjects, adhering to the Rules of Good Clinical Practice and the Declaration of Helsinki, Brazil 2013, as well as the current Spanish legislation on biomedical research projects, specifically Regulation 14/2007. Study data were collected and managed using REDCap electronic data capture tool^23,24^ hosted at HURyC.

### Participants

Adult patients (+ 18 years) diagnosed with cancer and experiencing pain (numeric scale VAS > 4/10) related to their oncologic process (including pain treatment) were included in the study. To participate, patients were required to possess a smartphone or a compatible electronic device and the ability to utilize a mobile application after receiving a brief training session. Patients with non-oncologic pain were excluded from the study. All patients underwent treatment in the radiation oncology department of the HURyC. Informed consent was obtained from all patients.

### Study procedures

Prior to entering the study, patients were informed of the application’s existence and their potential participation. Upon signing the informed consent form, patients were assisted in installing the mobile application and provided with an informative leaflet detailing its usage. For each day with active alerts, telephone-based consultations were conducted to adjust medication. All other aspects of medical care were maintained in accordance with clinical practice, regardless of the application’s utilization. Clinical practice face-to-face visits were scheduled at the start of treatment and throughout follow-up at weeks 2, 4, 6, 8, and at months 3, 6, and 12 post-treatment.

**Fig. 1:**
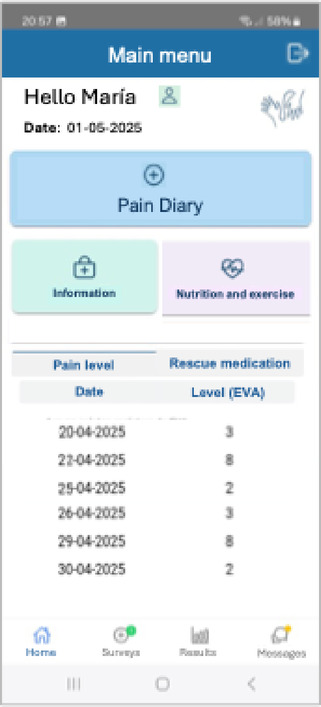
Home interface of the Accompain application.

Patients were provided with a mobile app (Accompain) designed specifically for this study and available both for Android and IOS devices. Patients used Accompain to record daily pain and medication data. Quality of life and adverse effect questionnaires were also collected. Attending physicians received automatic alerts whenever the pain level or the number of daily rescue medications were higher than the configured thresholds. Doctors contacted the patient by phone or using the app messaging module. The main page of the application is shown in Fig. 1. The recruitment period for the study lasted for 24 months.

### Measurements

Adherence was defined as having registered at least one of the variables collected in the application in relation to all the patients who logged in. The evolution of pain recorded by patients was obtained through the VAS questionnaires. The evolution of quality of life was assessed with the European Organization for Research and Treatment of Cancer Quality of Life Questionnaire C-30 (EORTC QLQ-C30)^25^. The pain interference was recorded with the BPI questionnaire. The symptoms were monitored using the Common Terminology Criteria for Adverse Events version 5.0 (CTCAE v5.0) (National Cancer Institute, Bethesda, Maryland)^26^. The frequency of rescue medication administration and the number of alerts were also analyzed. Six types of alerts were defined: (1) ≥ 30% pain intensity increase between two consecutive instances occurred in a period of no more than 3 days; (2) ≥ 30% rescue medication increase between two consecutive instances occurred in a period of no more than 3 days; (3) pain intensity > 7; (4) ≥ 30% pain intensity increase (pain intensity between 3 and 8) between two consecutive instances occurred in a period of no more than 3 days; (5) more than 6 rescue medications taken; and (6) ≥ 30% rescue medication increase (rescue medications taken between 3 and 8) between two consecutive instances occurred in a period of no more than 3 days. Time points for data analysis are: baseline, 15, 30, 45, and 60 days.

### Statistical analysis

The evolution of pain was analyzed using a One-Way ANOVA to compare pain intensity at each time point, and a Mixed-effects Model (REML) to compare pain intensity at each time point between males and females, and between patients with metastases compared to patients without metastases (localized tumors). Kruskal-Wallis and Mann-Whitney tests were used to estimate the effect of age range and gender on mean pain. The Kruskal-Wallis test is used to evaluate mean pain at different time points since the inclusion of each patient in the study. Descriptive statistics were reported as means ± SDs in the case of continuous variables and absolute and relative numbers for categorical variables. Alerts were analyzed using a Kruskal-Wallis test, and a Two-way ANOVA to analyze the effect of gender and type of tumor on the odds of triggering an alarm. Quality of life was analyzed using a Kruskal-Wallis test, and a Two-way ANOVA to analyze the effect of the type of tumor. Pain interference was analyzed using a Mixed-effects Model (REML) to compare interference in general activity, mood, and sleep at each time point. Daily rescue medication was assessed using a Kruskal-Wallis test to determine the evolution at different time points. The statistical analysis is conducted using GraphPad Prism (version 10.3.1; GraphPad Software).

## Results

From August 2022 to October 2024, a total of 87 patients (53 women) received information about the study and agreed to participate. Median age was 63 years (27–94). Fifty-one patients used the mobile app (59%); thirteen patients logged into the app but did not record information (15%); the remaining twenty-three never logged into the app, and they were considered enrollment dropouts (26%). According to our definition of adherence, enrollment dropouts were excluded from the adherence analysis, resulting in an adherence to pain recording of 79.69% (51/64).

Demographic characteristics of patients are in Table 1. Statistically significant differences were observed between users and dropout groups in terms of gender, with a higher proportion of male patients in the dropout group. Of the users group, 39.29% deceased within three months of enrollment. At the conclusion of the study, a total of 42 deaths were recorded, with a mean time from study entry to death of 5.61 ± 4.48 months. The distribution of deaths was more pronounced in the dropout group (55.56% vs. 43.13%).

**Table 1:** Demographic and clinical characteristics for users and dropout groups.

	All participants (*n* = 87)	Users (*n* = 51)	Enrollment Dropouts (*n* = 36)	*p* value
Sex				0.0154
Male	34 (39.08%)	14 (27.45%)	20 (55.56%)	0.1449
Female	53 (60.92%)	37 (72.55%)	16 (44.44%)	0.6968
Age	63 (27–94)	62 (33–94)	63.5 (28–89)	0.8018
Male	65 (27–81)	64 (50–79)	66 (28–81)	0.7826
Female	62 (33–94)	62 (33–94)	59.5 (48–89)	0.5220
Type of tumor				0.7583
Metastases	44 (50.57%)	27 (52.94%)	17 (47.22%)	0.1317
Localized tumors	43 (49.43%)	24 (47.06%)	19 (52.78%)	0.4458
Deceased patients at study completion	42 (48.27%)	22 (43.13%)	20 (55.56%)	0.3556
Male	22 (52.38%)	9 (40.91%)	13 (65.00%)	0.9831
Female	20 (47.62%)	13 (59.09%)	7 (35.00%)	0.5641

Data are in number (%), or median (range).

Patients recorded on average follow-up data for 53.00 ± 71.43 days. On average, patients started using the application (recorded the first VAS questionnaire) 1.68 days since the inclusion in the study.

### Adherence analysis

Each patient completed a mean of 43.96 ± 66.05 diary entries, including the VAS, the CTCAE Symptoms, and Medications, and a mean of 2.92 ± 4.05 standardized questionnaires. The distribution of completed questionnaires by patients is shown in Table 2. In the period of 15 days since the inclusion in the study, 94.12% of patients completed VAS questionnaires. After more than 60 days since the inclusion, the percentage had decreased to 35.29%. Patients with localized tumors (including anal-rectal cancer, breast cancer, head and neck cancer, and others) registered a higher number of VAS entries daily than patients with metastases (1.35 ± 0.83 vs. 1.09 ± 0.33). At 3 months post-inclusion, 33.33% of patients continued using the app (70.59% completed VAS entries); at 6 months, 5.88% of patients used the app (33.33% completed VAS entries); and 1-year post-inclusion, 3.92% of patients continued using the app (100% completed VAS entries).

**Table 2:** Distribution of completed questionnaires during the study.

	Total	Questionnaires per patient^a^	Patients (*n*)	Degree of compliance
Standardized questionnaires	146 (6.17%)	2.92 ± 4.05	25	
BPI	74 (50.68%)	1.49 ± 2.15	21	3.92%
EORTC QLQ-C30	72 (49.32%)	1.43 ± 1.93	25	0.00%
Daily questionnaires	2219 (93.83%)	43.96 ± 66.05	50	
VAS	1372 (61.83%)	27.16 ± 38.53	50	58.82%
CTCAE Symptoms	72 (3.24%)	1.45 ± 1.94	25	0.00%
Medication	775 (34.93%)	15.35 ± 30.28	38	33.33%
Follow-up days	765	53.00 ± 71.43	51	

Data are in number (%), or mean ± SD.

^a^Mean ± SD of questionnaires per patient was calculated considering the 51 users.

### Evolution of pain control

**Fig. 2:**
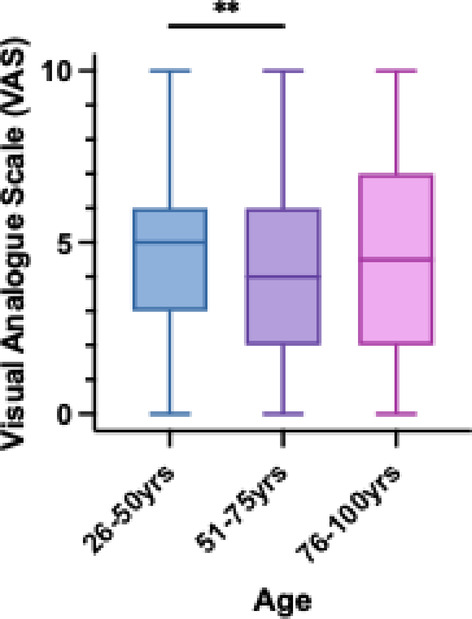
Mean pain according to Visual Analogue Scale (VAS) across different age ranges. ** *P* < .01.

**Fig. 3:**
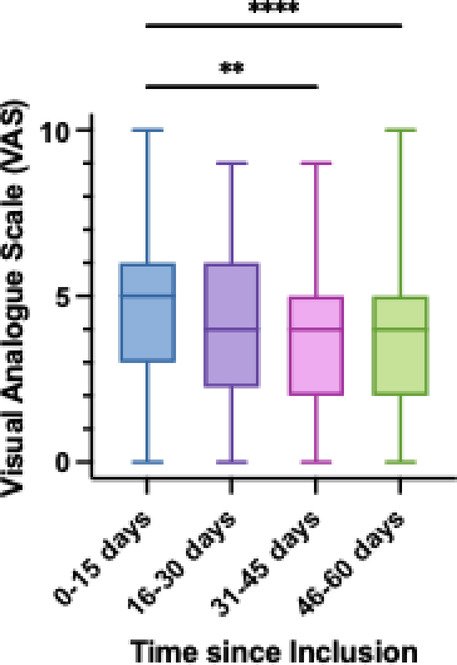
Evolution of pain according to Visual Analogue Scale (VAS) at different time points since the inclusion in the study. ** *P* < .01, **** *P* < .0001.

Figure 2 shows that younger patients (aged 26 to 50) experienced significantly more intense pain compared to older patients (aged 51 to 75). Patients aged 76 to 100 years present the highest variability. Figure 3 shows the temporal evolution of pain over time in relation to the time elapsed since inclusion in the study. Compared to the first two weeks (4.69 ± 2.53), patients reported less intense pain 31–45 days (3.87 ± 2.10, *p* < .01) and 46–60 days (3.57 ± 2.17, *p* < .0001). Analysis of the EORTC QLQ-C30 questionnaire showed that patients with localized tumors exhibited significantly poorer health and general quality of life compared to those with metastasis (*p* < .0001). There was no significant difference in pain-related QOL between the two groups. Pain was the domain among the three with the lowest quality of life (*p* < .0001).

In the analysis of the BPI questionnaire, which includes the exact location of pain, it was observed that pain intensity in the anterior trunk region tended to increase after 45 to 60 days from inclusion in the study compared to 0–15 days (*p* < .01); while pain in the posterior trunk region and the extremities of the body remained relatively stable.

Among the patients who reported symptoms (*n* = 23), 100% reported fatigue. Most patients reported persistent fatigue throughout the day (65.21%), whereas 43.48% only suffered fatigue during the afternoon and 21.74% in the morning. The overall intensity of fatigue was moderate (69.56%), mild (52.17%), and severe (8.69%). Additionally, 43.48% of the patients experienced headaches, 34.78% experienced dizziness and diarrhea, 60.87% experienced nausea, 13.04% experienced vomiting, 30.43% experienced constipation, and 65.22% presented other symptoms, among which vertigo, blurred vision, pruritus, low-grade fever, and anal bleeding were the most frequently described.

**Fig. 4:**
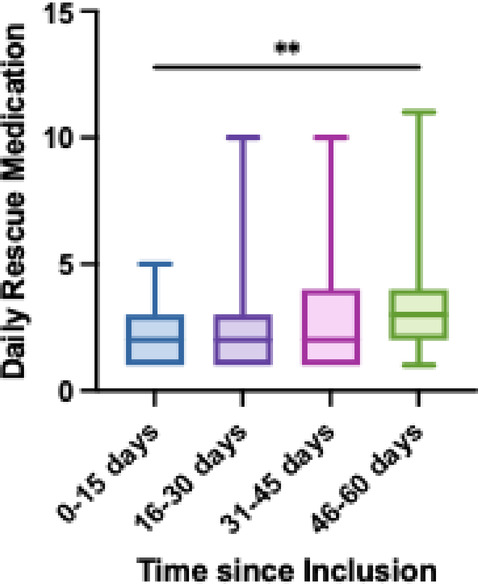
Changes in daily rescue medication at baseline, 15, 30, 45, and 60 days. ** *P* < .01.

Patients with metastases recorded a mean of 3.19 ± 2.33 rescues per day, and patients with a localized tumor had a mean of 2.26 ± 1.37 rescues per day (*p* < .0001). In total, 1386 rescues were recorded through the application. The distribution of daily rescue medication taken since the inclusion in the study is shown in Fig. 4. The most frequent rescue medication was metamizole 575 mg (World Health Organization analgesic ladder step 1), followed by paracetamol 1 g (step 1), and transmucosal fentanyl 100 mcg (step 3). Patients also recorded transmucosal fentanyl, paracetamol and ibuprofen (step 1) in other doses. Other medication added was dexketoprofen, transmucosal fentanyl by trade name (without specifying dosage), dexamethasone and naproxen. Some patients included basal medication in the rescue medication questionnaires, such as the fentanyl patches or extended-release morphine capsules or tabs. Patients used the free text feature to communicate medication discontinuations or their stay in the emergency department.

The system generated 542 notifications: 244 pre-alerts and 298 alerts sent to doctors’ professional emails from 27 patients. On average each of the 27 patients generated 3.55 ± 8.53 notifications. Patients with metastases registered the highest number of alerts for severe pain (VAS > 7), with a total of 108 alerts from 13 patients. The distribution of alerts by week, according to tumor type, shows that patients with metastases have a higher frequency of alerts for VAS > 7 (73.97%) compared to patients with localized tumors. Patients with metastases accounted for 81.25% of the 16 patients who received alerts for VAS > 7, and 55.55% of the 18 patients with alerts for VAS increase greater than 30% in the last three days. Patients with metastases also have a higher proportion of alerts (47.88%) compared to patients with localized tumors. Daily rescue medication report increased 46 to 60 days after inclusion in the study compared to two weeks after the inclusion (*p* < .01).

No patient required hospital admission or invasive procedures for pain control. Following the implementation of additional alerts to clinical practice, eleven face-to-face visits were scheduled. During the study period, 139 telephone-based consultations were conducted to assess the prescribed medication for each patient with active alerts (2.53 ± 3.89 alerts per week). In cases where a medication change was made, the patient was not contacted again for alerts generated within 24 h of the change. The consultations were carried out by the doctor responsible for the patient.

### Dropout analysis

Of the Enrollment dropout group (*n* = 36), 10% of the patients deceased during the first three months after the inclusion in the study and the mortality was of 55,56% at the end of the study. We contacted the alive dropout users (16 patients) to evaluate the reasons for not using the application. We contacted nine of the twenty-three patients who did not log in, and seven of the thirteen who logged in but did not enter any data. The main reported reasons were: they did not understand how to use the application (8 patients); they did not need the app since their symptoms were under control (5 patients); the app reminded them too much of their disease (2 patients); and one patient found it overwhelming.

**Fig. 5:**
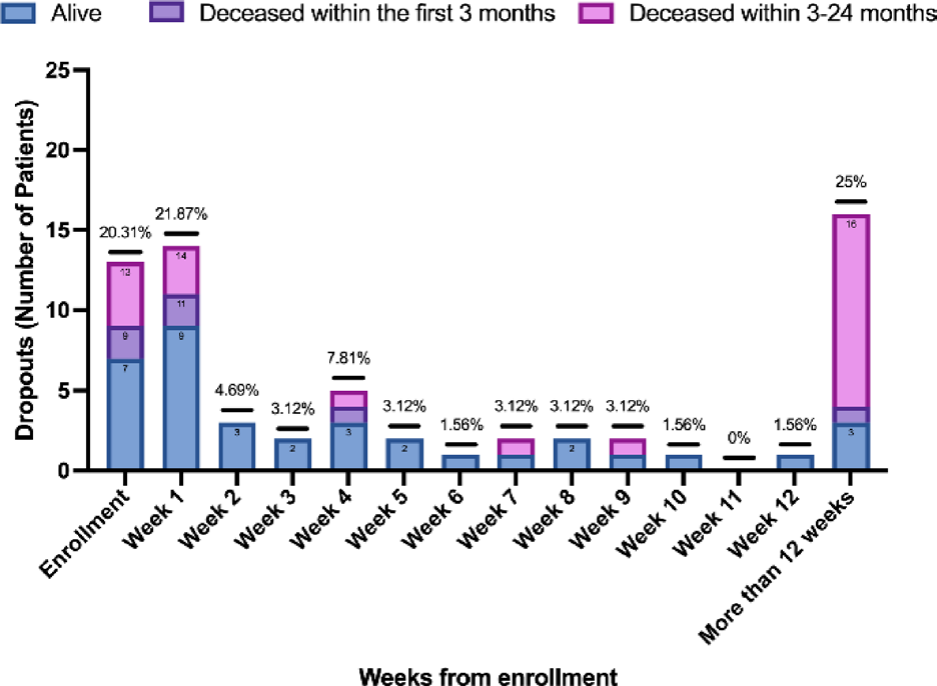
Dropout rates over the study time points.

Figure 5 shows the dropout rate over different study time points. Dropout was most pronounced in those patients who logged in but did not record any data (enrollment), in the first week and after more than 12 weeks since the enrollment. Most of the patients (16/20) who stopped using the app after 12 weeks deceased in that period.

## Discussion

The present validation study has demonstrated the feasibility of pain monitoring using a mobile application. Pain control in cancer has a relevant impact on patient’s quality of life^27^, but adequate pain management requires pain monitoring that is limited by the frequency of medical visits^8^. Pain applications have been demonstrated to be feasible with adequate patient and healthcare professional acceptance^20–22^. Nevertheless, the specific elements that can enhance adherence and facilitate pain management are not fully elucidated.

Several studies demonstrated an adherence to data recording of 76.8%^7^, 68.8 ± 38.1%^18^, 72%^28^ and 61.9%^10^, similar data to our study, in which adherence was 73.56%. However, we should consider that the measurement of adherence varies according to the study, and it is complex to establish correlations. Armbruster et al. categorized factors influencing adherence to cancer-related applications into three domains: sociodemographic variables (age, sex, educational level, emotional state), cancer-related factors (symptoms, stage, type of treatment), and other (comorbidities, personality traits)^29^.

Zhu et al. demonstrated a positive correlation between age and the duration of app usage (*p* = .003)^30^. Conversely, Lozano-Lozano et al. found that older age was associated with an increased risk of app abandonment (*p* = .001)^31^. Chung et al. observed that younger patients completed data collection more frequently^32^. In our study, the age distribution of patients who utilized the app was comparable to that of those who did not, 62 years (33–94) for those who used Accompain and 63.5 years (28–89) for those who did not, respectively (*p* = .8). The time of application usage exhibited high variability.

In terms of gender, Mikolasek et al. demonstrated that women exhibited higher adherence to continued app usage over time compared to men (*p* = .005)^33^. In our study, a greater proportion of women also used the app (14 men and 37 women, compared to 20 men and 16 women who did not use Accompain) (*p* = .01539). Regarding the pathologies of patients who did not use the application, no significant differences were observed between metastatic and non-metastatic patients, in line with the results reported by Greer et al.^34^.

Other features described in the literature that contribute to improved adherence include the personalization or adaptation of the application’s content to the individual needs of the user, reminders in the form of notifications, a user-friendly and technically stable design, and personalized support based on a systematic review^35^. All these factors were considered in the development of the application. In our study, it is noteworthy that after interviewing some patients who did not use the application, further efforts must be dedicated to user training. Of the dropout patients more than half of them died during the study, explaining to some extent the lack of adherence. The application was designed for use in different types of tumors, improving doctor-patient communication, data quality and patient health status assessment. It specifically focuses on psychological evaluation by introducing questions about the patient’s emotional state, which is not commonly found in applications of this type^36–39^.

Patients registered VAS and medication diaries more intensively than the standardized questionnaires, probably because they perceive a positive and higher impact in their treatment adjustments. Monitoring through Accompain has provided more patient information (2365 questionnaires) compared to face-to-face visits, facilitating patient evaluation and treatment adjustments before symptoms escalate and become more challenging to manage, as observed by Rau et al.^40^. Furthermore, the implementation of an alert system enabled rapid intervention on 298 occasions, with action ultimately being taken in 139 of these cases. The alert system represents an advancement from clinical practice, since it allows healthcare professionals to adjust the medication in between visits, which Vercell et al. remarks as one of the features lacking in most of the studies^41^.

The primary limitation of this pilot usability study is the relatively small sample size of patients to include in the analysis the outcomes segmented by gender, age, and pathology.

## Conclusion

Pain ambulatory monitoring enables remote assessment of patients’ clinical situations, impacting the management of pain by medical professionals. This study confirms the variability of patient adherence to health applications, being gender the factor that higher impacts in adherence. Pain monitoring is feasible, but further research is necessary to explore the clinical impact and develop novel methods to assist professionals in managing pain in oncology patients. Additional ways of engaging sicker patients and less IT-skilled users need to be developed, such as the inclusion of voice messages and natural language processing.

## Data Availability

Raw data for this study are not publicly available to preserve individuals’ privacy under the European General Data Protection Regulation. If necessary, data can be provided under certain conditions by Dr. C. de la Pinta.
